# Anemia among HIV-Infected Patients Initiating Antiretroviral Therapy in South Africa: Improvement in Hemoglobin regardless of Degree of Immunosuppression and the Initiating ART Regimen

**DOI:** 10.1155/2013/162950

**Published:** 2013-08-27

**Authors:** Simbarashe Takuva, Mhairi Maskew, Alana T. Brennan, Ian Sanne, A. Patrick MacPhail, Mathew P. Fox

**Affiliations:** ^1^Clinical HIV Research Unit, Department of Internal Medicine, School of Clinical Medicine, Faculty of Health Sciences, University of the Witwatersrand, Johannesburg 2041, South Africa; ^2^Health Economics and Epidemiology Research Office, Department of Internal Medicine, School of Clinical Medicine, Faculty of Health Sciences, University of the Witwatersrand, Johannesburg 2193, South Africa; ^3^Center for Global Health and Development, Boston University, Boston, MA 02118, USA; ^4^Department of Epidemiology, Boston University School of Public Health, Boston, MA 02118, USA

## Abstract

Among those with HIV, anemia is a strong risk factor for disease progression and death independent of CD4 count and viral load. Understanding the role of anemia in HIV treatment is critical to developing strategies to reduce morbidity and mortality. We conducted a prospective analysis among 10,259 HIV-infected adults initiating first-line ART between April 2004 and August 2009 in Johannesburg, South Africa. The prevalence of anemia at ART initiation was 25.8%. Mean hemoglobin increased independent of baseline CD4. Females, lower BMI, WHO stage III/IV, lower CD4 count, and zidovudine use were associated with increased risk of developing anemia during follow-up. After initiation of ART, hemoglobin improved, regardless of regimen type and the degree of immunosuppression. Between 0 and 6 months on ART, the magnitude of hemoglobin increase was linearly related to CD4 count. However, between 6 and 24 months on ART, hemoglobin levels showed a sustained overall increase, the magnitude of which was similar regardless of baseline CD4 level. This increase in hemoglobin was seen even among patients on zidovudine containing regimens. Since low hemoglobin is an established adverse prognostic marker, prompt identification of anemia may result in improved morbidity and mortality of patients initiating ART.

## 1. Background

Anemia has been shown to be the most frequent hematological abnormality in HIV-infected patients globally [1, 2]. Even among those initiating antiretroviral therapy (ART), anemia has been demonstrated to be a strong risk factor for disease progression and subsequent death [1–5] independent of CD4 count and viral load. In a large European cohort study, the presence of severe anemia at ART initiation was associated with a 13-fold increased risk of death [3]. In sub-Saharan Africa, which has the largest burden of HIV in the world, anemia is common as patients are more likely to be malnourished, have advanced immunosuppression, and have higher rates of comorbidities (especially tuberculosis and malaria) than those in high-income countries [6, 7]. Despite the public health importance of anemia, prospective data from sub-Saharan Africa on its impact are limited. Recent reports suggest that hemoglobin levels improve with ART [2, 3, 8]; however, few studies have documented the evolution of hemoglobin levels among patients on ART in resource-limited settings, and whether the effects on hemoglobin levels vary by ART regimen. Given the number of patients on ART in this region, understanding the role of anemia in HIV treatment is critical to developing strategies to improve survival and reduce morbidity on ART. 

The major cause of anemia is impaired erythropoiesis resulting from the release of inflammatory cytokines and decreased production of hematopoietic growth factors, coupled with malabsorption and impaired recycling of iron [9, 10]. Additionally, there are multiple other causes of anemia, which include nutritional deficiencies (iron, cobalamine, or folate deficiency), malignant bone marrow infiltration, bone marrow infection, and hemolysis [11–14]. Among patients initiating antiretroviral therapy, the use of zidovudine containing regimens has been associated with the incidence of anemia, and bone marrow toxicity has been postulated [2, 9, 10]. Amongst patients in an urban HIV clinic in Uganda, severe anemia improved with ART in the majority of patients. These findings suggested that baseline severe anemia should not be used as a criterion for avoiding the use of zidovudine in patients initiating ART in resource-limited settings [15, 16].

In this prospective cohort study conducted in Johannesburg, South Africa, we set out to determine the prevalence, incidence, and predictors of anemia among patients initiating first line ART. We also sought to explore whether the degree of immunosuppression at ART initiation or the initiating ART regimen (zidovudine versus other) impacted the magnitude of hemoglobin increase while on treatment.

## 2. Methods

### 2.1. Study Population

The Themba Lethu Clinic is one of the largest HIV care and treatment clinics in South Africa with over 22,000 patients initiated onto ART since 2004 [17]. We included ART naïve, nonpregnant HIV-infected patients ≥18 years of age, initiated on standard first-line ART at Themba Lethu from April 2004 to August 2009 with documented hemoglobin measurements. All patients had 24 potential months of follow-up time from ART initiation.

### 2.2. Study Procedures

At Themba Lethu, ART is provided according to the South African Department of Health Guidelines [18]. All patients are scheduled to have a full blood count at ART initiation, four months after initiation, and then every six months thereafter. The laboratory data are captured onto a longitudinal patient management electronic record. With the full blood count measurements, whilst clinicians read the results, only hemoglobin values are completely and accurately captured onto the system. Hence, in this study, measurements characterizing any anemia were not available. If initiated onto a zidovudine (AZT) containing regimen, a full blood count is done at initiation, monthly for the first three months on treatment, and then every six months thereafter. Only patients with a contraindication to stavudine (d4T) at ART initiation would be given AZT in place of d4T (i.e., pregnant women or patients receiving tuberculosis treatment). During the study period from April 2004 to August 2009, the 2004 South Africa National Department of Health HIV Treatment Guidelines were in force [18]. 

For all patients, person-time accrued from ART initiation until the date of the begining of (1) incident anemia; (2) last clinic visit; (3) loss to follow-up; (4) death; (5) transfer; or (6) completion of 24 months of follow-up. Loss to follow-up was defined as 4 months since last clinic visit. At Themba Lethu, mortality is ascertained via family or hospital report, active tracing, and linkage with the South African National Vital Registration Infrastructure Initiative. 

### 2.3. Definition of Anemia

We used the National Institutes of Health Division of AIDS definitions for Grading the Severity of Adult and Paediatric Adverse Events to define anemia [19]. Anemia was defined as a hemoglobin level of <10 g/dL. Incident anemia was defined as new-onset anemia during the 24 month follow-up period among patients who were not anemic at initiation of ART.

### 2.4. Statistical Analysis

We calculated the prevalence and incidence of anemia at ART initiation and during follow-up, respectively. Prevalence ratios (PR) and 95% confidence intervals (95% CI) for the association of baseline characteristics with prevalent anemia were estimated using modified Poisson regression. Cox proportional hazards models were used to evaluate the association between incident anemia and baseline (defined as 90 days before to 7 days after ART initiation) characteristics such as hemoglobin, CD4 count, body mass index (BMI), and WHO stage. We further examined overall changes in hemoglobin levels on treatment by stratifying results by CD4 count and ART regimen (AZT versus non-AZT containing regimen) at treatment initiation.

### 2.5. Ethics Approval

Use of Themba Lethu Clinic data was approved by the Human Research Ethics Committee (medical) of the University of Witwatersrand, and approval for analysis of anonymized data was approved by the Boston University Institutional Review Board.

## 3. Results

### 3.1. Patient Characteristics at Initiation of ART

Between April 2004 and August 2009, 10,369 patients fulfilled the inclusion criteria. Among these, 102 (1%) did not have hemoglobin measurements, and 8 patients had abnormally high hemoglobin levels (>30 g/dL) at ART initiation leaving 10,259 patients for analysis. Excluded patients were largely similar in demographic and clinical characteristics from those who were included in analyses. The majority of patients were females (68%), with a median age of 36 years (IQR 31–43) and initiated on stavudine-lamivudine-efavirenz (89.4%) (Table 1). The cohort was immunosuppressed at ART initiation, with a median CD4 count of 82 cells/mL (IQR 30–151), 45.7% with WHO stage III/IV, and 19.9% with a tuberculosis diagnosis.

### 3.2. Prevalent Anemia

Overall prevalence of anemia at ART initiation was 25.8% (95% CI: 25.0%–26.7%). In multivariable analysis, females (PR: 1.77; 95% CI: 1.64–1.90) and younger patients (18–35 versus >55; PR: 1.59; 95% CI: 1.19–2.11) were more likely to be anemic at ART initiation (Table 2), while patients initiated onto AZT-based regimens (versus d4T-based) were less likely to be anaemic (PR: 0.56; 95% CI: 0.42–0.75) at the start of treatment. Only 3.1% (*n* = 322) of the patients were initiated on to AZT-based regimens. Of note, among these 322 patients initiated onto AZT-based regimens, 47 patients corresponding to 14.6% already had hemoglobin levels of less than 10 g/dL at baseline. Additionally, we found that those with a WHO III/IV versus I/II (PR: 1.96; 95% CI: 1.81–2.12), patients with a low CD4 count (<50 versus >200 cells/mL; PR: 1.48, 95% CI: 1.28–1.73), and those with tuberculosis at initiation (PR: 1.14, 95% CI 1.06–1.22) were more likely to have anemia at ART initiation. 

### 3.3. Incident Anemia

A total of 7,612 patients had normal hemoglobin levels at baseline. The incidence rate for anemia among these patients was 3.1/1000 person-years (PY). Incidence of anemia was the highest in the first 3 months after ART initiation at a rate of 34.7/1000 PY and decreased to 16.7/1000 PY between 3 and 6 months on treatment. This incidence was then fairly stable over the remaining 18 months of follow-up, between 7.7 and 8.4/1000 PY. In adjusted analyses (Table 1) female gender (adjusted hazards ratio (aHR): 1.78; 95% CI: 1.45–2.19), lower BMI (aHR: 1.70; 95% CI: 1.35–2.14), WHO III/IV (aHR: 1.24; 95% CI: 1.02–1.51), and lower CD4 count (<50 versus >200 cells/mL, aHR: 1.72; 95% CI: 1.18–2.52) were associated with increased incident anemia over the follow-up period. 

We found that AZT use was associated with over twice the rate of developing anemia during follow-up (aHR: 2.19; 95% CI: 1.50–3.20). In a sensitivity analysis to decrease measurement error, we further restricted the analysis to patients without an indication for AZT. Our hazard ratios were similar though the association between AZT and incident anemia was slightly attenuated, from 2.19 to 1.88 (95% CI: 1.17–3.03). 

### 3.4. CD4 Count and ART Regimen Category and Change in Hemoglobin Levels

**Figure 1:**
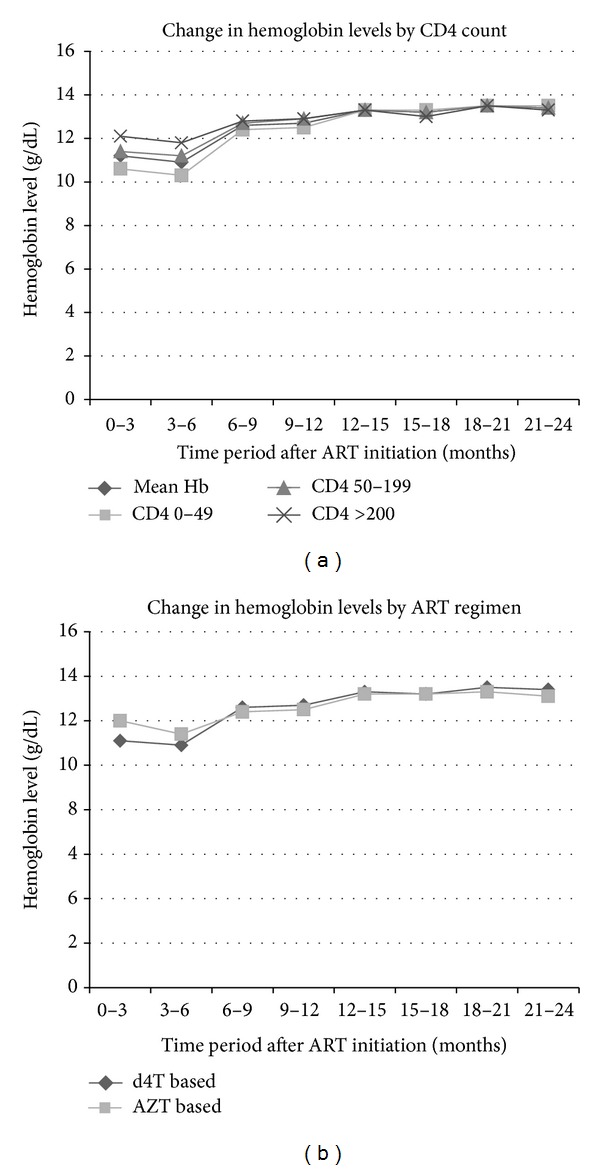
Change in hemoglobin levels by level of immunosuppression and initiating ART regimen amongst patients at the Themba Lethu Clinic in Johannesburg, South Africa (*n* = 10,259). Abbreviations: AZT: zidovudine; d4T: stavudine; ART: antiretroviral therapy.

Among the 10,259 patients initiating ART, hemoglobin increased over 24 months on ART (Figure 1). Between 0 and 3 months on ART, mean hemoglobin was 11.2 g/dL but increased to 13.6 g/dL between 21 and 24 months. Regardless of CD4 at ART initiation, hemoglobin levels increased over time. Between 0–6 months on ART, the magnitude of hemoglobin increase was linearly related to CD4 count. However, between 6 and 24 months on ART, hemoglobin levels showed a sustained overall increase, the magnitude of which was similar regardless of baseline CD4 count. At ART initiation, patients on AZT containing regimens had higher mean hemoglobin (mean 12.0 g/dL, sd 2.0) compared to those initiated onto regimens without AZT (mean 11.4 g/dL, sd 2.2). From 6 and 24 months on ART, mean hemoglobin increased for both regimen categories. For patients on AZT containing regimens, mean hemoglobin increased from 12.3 g/dL to 13.3 g/dL, and for patients on regimens without AZT, mean hemoglobin increased from 12.6 g/dL at initiation to 13.4 g/dL by 24 months.

## 4. Discussion

This is a study of patients under routine public-sector HIV care in Johannesburg, South Africa, over a quarter of patients presented at treatment initiation with anemia (Hb < 10 g/dL). This is slightly higher than previous estimate of 18% of prevalence of anemia among HIV-infected patients in Southern Africa but in line with estimates for other regions of Africa (between 24% and 37%) [20]. We estimate the incidence of anemia at 3.1/1000 person-years and found it to be the highest in the first 6 months after ART initiation. Since hemoglobin measurements are simple and inexpensive, monitoring hemoglobin levels over the first 6 months on ART among patients at high risk may allow for improved detection of HIV-related anemia. It may also lead to detection of related underlying comorbid illnesses manifesting as anemia associated with poor treatment outcomes [1–5]. Beyond 6 months, our data shows a lower incidence of anemia in support of current recommendations to measure hemoglobin levels every 6 months and if clinically indicated during this time [18].

In our data, incident anemia was associated with female gender, lower BMI, advanced WHO stage, and lower CD4 count. The association of anemia with lower BMI is likely to reflect malnutrition, as a number of nutritional deficiencies contribute to anemia. The increased risk in anemia among patients with immunosuppression is in agreement with previous published reports [16, 21–24]. Being on an AZT containing regimen was also a risk factor for developing anemia. Studies have shown that AZT can inhibit bone marrow activity, resulting in decreased production of blood cells and platelets; previous research has demonstrated its association with incident anemia [16, 22, 23, 25, 26]. 

The present study also showed that over 24 months of follow-up, hemoglobin increased for patients initiating ART, similar to findings from resource-rich environments [2, 3]. This increase in hemoglobin was seen regardless of ART regimen (both AZT- and d4T-based regimens). This observation is in contrast to a meta-analysis of six clinical trials conducted in high-income settings that reported decreases in hemoglobin levels during 48 weeks of follow-up among patients initiating AZT containing regimens [10]. However, in a cohort of HIV-infected patients accessing ART from a rural hospital in Tanzania, the majority of patients who were anemic at the time of ART initiation had significant hemoglobin increase over the initial 12 months of ART, and in this study, zidovudine-containing initial regimen was an independent predictor of persistent anemia [8]. This pattern of improving hemoglobin level despite initiating an AZT-containing regimen is also similar to observations in some South African [27] and Ugandan cohorts [15]. 

Our study has several strengths including large number of clinical events owing to the relatively large sample size. Also, we use routine clinical data collected from a real life clinical setting. However, the findings of this study should be considered alongside its limitations. We did not collect comprehensive information of clinical conditions that may be possible underlying causes of anemia, resulting in unmeasured confounding of the risk factors for prevalent and incident anemia. Additionally, loss to follow-up may have led to selection bias, as outcome cannot be determined in patients lost or those who have died. 

In conclusion, we observed a high prevalence of anemia among HIV-infected patients initiating first-line ART in Johannesburg, South Africa. Over 24 months of follow-up, incidence of anemia was the highest in the first 6 months after ART initiation, but, overall, during follow-up, this remained low. Risk factors for prevalent and incident anemia were similar to those reported elsewhere. Our results further support findings that ART improves hemoglobin and this is seen regardless of regimen type (AZT containing versus non-AZT containing) and the degree of immunosuppression. Hemoglobin measurement is an inexpensive marker for HIV disease progression and should be measured more frequently in patients at risk for anemia in the first 6 months after initiation of ART. Our study suggests that ART improves hemoglobin levels regardless of magnitude of immunosuppression and ART initiating regimen.

## Figures and Tables

**Table 1:** Baseline characteristics among patients initiating first-line ART and the risk factors for incident anemia at the Themba Lethu Clinic in Johannesburg, South Africa.

	Baseline characteristics				Risk factors for incident anaemia			
	Overall (*n* = 10259)		Anaemia (*n* = 2647)		Univariate analysis		Multivariate analysis	
	No.	%	No.	%	cHR	95% CI	aHR	95% CI
Sex								
Male	3904	38	738	18.9	1.00	Referent	1.00	Referent
Female	6355	62	1909	30	1.56	1.28–1.92	1.78	1.45–2.19
CD4 count (cells/mL)								
0–49	3639	35.5	1206	33.1	1.79	1.23–2.59	1.72	1.18–2.52
50–199	5754	56.1	1298	22.6	1.15	0.80–1.66	1.17	0.89–1.68
>200	866	8.4	143	16.5	1.00	Referent	1.00	Referent
BMI (kg/m^2^)								
<18.5	2039	19.9	823	40.4	1.74	1.39–2.17	1.70	1.35–2.14
≥18.5	8220	80.1	1824	22.2	1.00	Referent	1.00	Referent
Age (years)								
18–35	4483	43.7	1250	23.9	1.24	0.71–2.19	—	—
36–55	5397	52.6	1333	27.5	1.12	0.64–1.96	—	—
>55	379	3.7	64	13.1	1.00	Referent		
WHO stage								
I and II	5567	54.3	899	16.1	1.00	Referent	1.00	Referent
III and IV	4692	45.7	1748	37.2	1.40	1.16–1.68	1.24	1.02–1.51
ART regimen								
d4T based	9937	96.9	7337	96.4	1.00	Referent	1.00	Referent
AZT based	322	3.1	275	3.6	2.03	1.39–2.96	2.19	1.50–3.20
TB diagnosis								
No	8223	80.1	1833	22.3	1.00	Referent		
Yes	2036	19.9	814	40	1.41	1.12–1.77	—	—

Abbreviations: cHR: crude hazard ratio, aHR: adjusted hazard ratio, HR: hazard ratio, 95% CI: 95% confidence interval, ART: antiretroviral therapy, Hb.: hemoglobin, BMI: body mass index, EFV: efavirenz, AZT: zidovudine, NVP: nevirapine, IQR: interquartile range, TB: Tuberculosis. 95% CI: 95% confidence interval; HR was estimated using Cox proportional hazards regression models.

**Table 2:** Association of baseline characteristics with prevalent anemia among 10,259 patients initiating first-line ART at the Themba Lethu Clinic in Johannesburg, South Africa.

	Univariate analysis		Multivariate analysis	
	Crude PR	95% CI	Adjusted PR	95% CI
Sex				
Male	1.00	Referent	1.00	Referent
Female	1.59	1.47–1.71	1.77	1.64–1.90
CD4 count (cells/mL)				
0–49	2.01	1.72–2.35	1.48	1.28–1.73
50–199	1.37	1.17–1.60	1.19	1.02–1.38
>200	1.00	Referent	1.00	Referent
BMI (kg/m^2^)				
<18.5	1.82	1.70–1.94	1.57	1.46–1.70
≥18.5	1.00	Referent	1.00	Referent
Age (years)				
18–35	1.83	1.37–2.45	1.59	1.19–2.11
36–55	2.10	1.58–2.80	1.54	1.16–2.04
>55	1.00	Referent	1.00	Referent
WHO stage				
I and II	1.00	Referent	1.00	Referent
III and IV	2.31	2.15–2.48	1.96	1.81–2.12
ART regimen				
d4T in regimen	1.00	Referent	1.00	Referent
AZT in regimen	0.56	0.42–0.74	0.56	0.42–0.75
TB diagnosis				
No	1.00	Referent	1.00	Referent
Yes	1.79	1.68–1.92	1.14	1.06–1.22

Abbreviations: PR: prevalence ratio, 95% CI: 95% confidence interval, ART: antiretroviral therapy, BMI: body mass index, EFV: efavirenz, 3TC: lamixudine, d4T: stavudine,  AZT: zidovudine, NVP: nevirapine, IQR: interquartile range, TB: tuberculosis.
